# Supplementary material for: Advances in Vaporized Hydrogen Peroxide Reusable Medical Device Sterilization Cycle Development: Technology Review and Patent Trends
Source: Microorganisms. 2023 Oct 15;11(10):2566. doi: 10.3390/microorganisms11102566 (PMC10608877; doi:10.3390/microorganisms11102566)
Supplement: Supplementary file 1 [file microorganisms-11-02566-s001.zip › microorganisms-2641499-supplementary.pdf]

Supplementary information

Advances in vaporized hydrogen peroxide reusable medical device sterilization cycle development: Technology review and patent trends

M.R. Karimi Estahbanati

Université Laval, Chemical engineering department, 1065 avenue de la Médecine, Quebec, QC, G1V 0A6, Canada

Table S1. Summary of the parameters of Flexible cycle of V-PRO® maX 2 sterilizer.

**Phase**		**Initial pressure (Torr)**	**Final pressure (Torr)**	**Duration (min)**	**Comment**
**Moisture check**	Evacuation and moisture verification	-	0.4	-	-
	Conditioning	0.4	< 0.4	-	If moisture available
	Moisture re-verification	-	-	-	If moisture available. Abort if fails.
**Sterilize**	H_2O_2 injection	0.4	6.3 - 15	-	-
	Hold segment	-	-	2	-
	Air injection	-	500	-	-
	Hold segment	500	500	1	-
	Pressure reduction	500	0.4	-	-
	Re-evacuation and re-sterilization	-	-	-	3 repeats
**Aerate**	Aeration	1	-	-	-

Table S2. Summary of 510(k) premarket notification clearances of STERRAD 100NX and NX as well as V-Pro Max and S series sterilizers.

Manufacturer	Device name	510(k) number	Decision date	Modification
ASP	Sterrad Nx With Allclear Technology	K220404	05/16/2022	An expansion of existing claims. No cycle modification.
	STERRAD® 100NX Sterilizer with ALLClear Technology	K212174	10/09/2021	An expansion of existing claims. No cycle modification.
	Sterrad NX Sterilizer	K162007	02/16/2017	Vacuum pump and associated mounting components modification. No cycle modification.
	Sterrad® NX Sterilizer With Allclear Technology	K160818	09/27/2016	Addition of Load Conditioning Feature. Do not modify the existing sterilization cycles.
	Sterrad 100NX Sterilizer with Allclear Technology	K160903	09/26/2016	Addition of Load Conditioning Feature. Do not modify the existing sterilization cycles.
	Sterrad NX Sterilizer, Sterrad 100NX Sterilizer	K151725	01/13/2016	Microprocessor module change with no cycle modification.
	Sterrad NX Sterilizer, Sterrad 100NX Sterilizer	K142454	04/03/2015	Software revision and upgraded CPU. No cycle modification.
	Sterrad 100NX Sterilizer Duo Cycle	K111377	09/26/2012	Addition of DUO Cycle.

	Sterrad 100NX Sterilizer Express Cycle	K092622	03/04/2011	Addition of Express Cycle.
Steris	V-Pro Max 2 Low Temperature Sterilization System, V-Pro S2 Low Temperature Sterilization System	K222849	09/30/2022	Same control but using slightly modified boards. No cycle modification.
	V-Pro Max 2 Low Temperature Sterilization System, V-Pro Max Low Temperature Sterilization System	K222093	09/09/2022	Modification of indications for use. No cycle modification.
	V-Pro Max 2 Low Temperature Sterilization System, V-Pro S2 Low Temperature Sterilization System	K222543	08/25/2022	Same control but using refurbished/slightly modified boards. No cycle modification.
	V-Pro S2 Low Temperature Sterilization Systems	K190917	05/09/2019	Modification of indications for use. No cycle modification.
	V-Pro Max 2 Low Temperature Sterilization System, V-Pro Max Low Temperature Sterilization System	K190103	04/05/2019	Modification of indications for use for the Lumen cycle. No cycle modification.

V-Pro S2 Low Temperature Sterilization System	K182568	01/03/2019	Modification of indications for use. No cycle modification.
V-Pro Max 2 Low Temperature Sterilization System	K172754	02/09/2018	Addition of the Fast Non Lumen Cycle.
V-Pro Max Low Temperature Sterilization System	K172319	01/18/2018	Modifications to labeling. No cycle modification.
V-Pro Max Low Temperature Sterilization System	K162413	03/31/2017	Remove labeling restrictions. No cycle modification.
V-Pro® Max Low Temperature	K160433	07/06/2016	Indications differ slightly. No cycle modification.
V-Pro Max Low Temperature Sterilization System	K131120	07/16/2013	Indications of Lumen Cycle updated. No cycle modification.
V-Pro Max Low Temperature Sterilization System	K120632	06/26/2012	Modification of Lumen Cycle claims. No cycle modification.
Amsco V-Pro Max Low Temperature Sterilization System	K112813	12/01/2011	Modification of Lumen Cycle claims. No cycle modification.

Amsco V-Pro Max Low Temperature Sterilization System	K112760	11/22/2011	Modification of Lumen Cycle claims. No cycle modification.
Amsco V-Pro Max Low Temperature Sterilization System	K102330	08/12/2011	Introduction of V-Pro Max by addition of Flexible Cycle.
